# Circulating glucuronic acid predicts healthspan and longevity in humans and mice

**DOI:** 10.18632/aging.102281

**Published:** 2019-09-26

**Authors:** Andrew Ho, Jonah Sinick, Tõnu Esko, Krista Fischer, Cristina Menni, Jonas Zierer, Maria Matey-Hernandez, Kristen Fortney, Eric K. Morgen

**Affiliations:** 1 BIOAGE, Richmond, CA 94804, USA; 2 Estonian Genome Center, University of Tartu, Tartu 51010, Estonia; 3 Institute of Mathematics and Statistics, University of Tartu, Tartu 50409, Estonia; 4 Department of Twin Research, Kings College London, London SE1 7EH, United Kingdom

**Keywords:** glucuronic acid, glucuronate, mortality, aging, lifespan

## Abstract

Glucuronic acid is a metabolite of glucose that is involved in the detoxification of xenobiotic compounds and the structure/remodeling of the extracellular matrix. We report for the first time that circulating glucuronic acid is a robust biomarker of mortality that is conserved across species. We find that glucuronic acid levels are significant predictors of all-cause mortality in three population-based cohorts from different countries with 4-20 years of follow-up (HR=1.44, p=2.9×10^-6^ in the discovery cohort; HR=1.13, p=0.032 and HR=1.25, p=0.017, respectively in the replication cohorts), as well as in a longitudinal study of genetically heterogenous mice (HR=1.29, p=0.018). Additionally, we find that glucuronic acid levels increase with age and predict future healthspan-related outcomes. Together, these results demonstrate glucuronic acid as a robust biomarker of longevity and healthspan.

## INTRODUCTION

Glucuronic acid is a key metabolite of glucose involved in the detoxification of xenobiotic compounds [1–6]. Many of these exogenous compounds, which include pollutants and drug metabolites, undergo hepatic glucuronidation, in which they are conjugated to glucuronic acid via the enzymatic action of UDP-glucuronosyltransferases [7, 8]. This chemical modification increases solubility in bile, facilitates urinary excretion, and is a key step in the phase II metabolism of these compounds required for their effective clearance from the body [9]. However, enzymes known as β-glucuronidases cleave these conjugates, thereby counteracting this detoxification process [10], as well as steroid hormone conjugates, thereby altering steroid metabolism [11]. Around a quarter of resident bacterial species in the human gut produce β-glucuronidase [12–14], which has been directly linked to increased xenobiotic-induced toxicity rescuable by inhibition of the enzyme [15–17]. Another vital role of glucuronic acid is as a constituent of proteoglycans, a diverse class of glycosylated proteins known primarily as components of the mammalian extracellular matrix [18, 19], where glucuronic acid may confer increased rigidity [20]. These proteoglycans are degraded as a part of tissue remodeling by endogenous lysosomal β-glucuronidase, which, like its bacterial analogue, cleaves glucuronic acid moieties through hydrolysis [21].

The factors influencing circulating levels of glucuronic acid are poorly characterized. However, orally ingested glucuronic acid has been shown to raise serum glucuronic acid levels within an hour, suggesting that it is readily absorbed into the bloodstream [22]. The ingestion of glucuronide conjugates also increases glucuronic acid levels, likely through the absorption of glucuronic acid liberated in the gastrointestinal tract by β-glucuronidases [23]. Elevated serum levels of glucuronic acid have been reported in human studies of diabetes, hepatocellular carcinoma, hepatitis, cirrhosis, and obstructive jaundice [24–28]. These findings are not unambiguous, as subsequent work reported contradictory associations with hyperglycemia and hepatitis [25, 29, 30]. Finally, a recent metabolomic study of patients with cirrhosis identified glucuronic acid as a biomarker of disease severity and future mortality [31].

In the present study, we performed untargeted profiling of circulating metabolites in a large, population-based prospective human cohort study, followed by validation in two further prospective cohorts and one longitudinal mouse study. We report the novel discovery that circulating glucuronic acid is a robust, cross-cohort and cross-species predictor of all-cause mortality in healthy individuals, as well as a predictor in humans of chronological age and healthspan-related outcomes.

## RESULTS

### Circulating glucuronic acid levels predict all-cause mortality in humans

**Figure 1:**
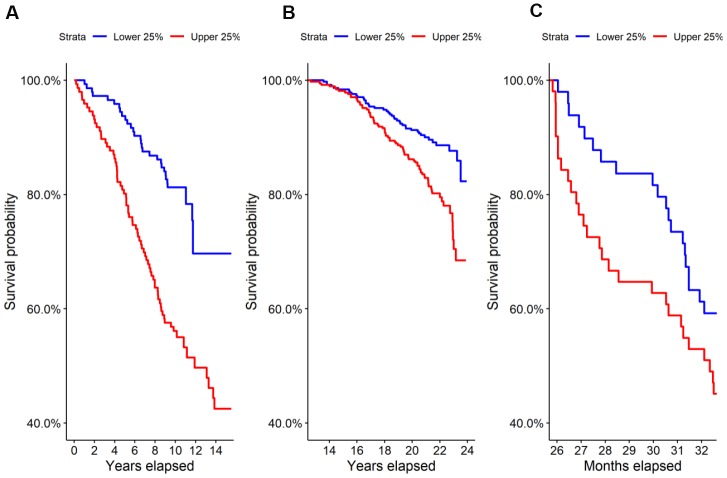
**Survival in the highest and lowest quartiles of glucuronic acid level.** Kaplan-Meier survival curves for the top vs. bottom quartiles of glucuronic acid level are plotted for (**A**) the Estonian Biobank discovery cohort, (**B**) the Framingham Offspring cohort, and (**C**) the longitudinal murine cohort.

We performed untargeted metabolomics on the Estonian Biobank cohort and found 69 of 569 identified metabolites to be predictive at FDR < 0.05 of all-cause mortality by Cox regression corrected for clinical covariates. Glucuronic acid ranked 9^th^ by p-value and was highly significant after correcting for multiple hypothesis testing (HR=1.44, p=2.9×10^-6^, FDR=5.0×10^-4^), with higher levels leading to shorter lives on average. A Kaplan-Meier survival curve comparing the top and bottom quartiles of glucuronic acid levels in the Estonian Biobank cohort is shown in Figure 1A and demonstrated a significant difference in survival between the curves (p=1.7×10^-6^).

We evaluated this association in two replication cohorts, the Framingham Offspring cohort and TwinsUK study cohort, by Cox regression corrected for clinical covariates. Demographic characteristics of these cohorts are shown in Table 1A. As in the Estonian cohort, we found that glucuronic acid levels predicted all-cause mortality in both the Framingham Offspring (N=1,479; HR=1.13, p=0.032; Kaplan-Meier curve in Figure 1B) and in the TwinsUK (N=1,761; HR=1.25, p=0.017) cohorts.

**Table 1:** Demographic and clinical characteristics by cohort.

**Characteristic**	**Estonian Biobank (discovery)**	**Framingham Offspring**	**TwinsUK**	**Estonian Biobank (secondary)**
Sample size	579	1,479	1,761	100
Deaths	189	306	47	0
Median time to death (years)	5.9 ± 3.8	18.3 ± 2.7	2.64 ± 2.1	n/a
Median follow-up time (years)	7.1 ± 2.7	21.5 ± 2.0	3.41 ± 2.39	n/a
Women (%)	69.8	53.0	100.0	50%
Age (years)	73.3 ± 2.7	53.7 ± 9.2	64.9 ± 8.4	41.7 ± 12.2
Body mass index	27.3 ± 4.3	22.5 ± 4.9	26.4 ± 4.9	26.2 ± 3.7
Systolic blood pressure (mm Hg)	140.1 ± 17.2	125.4 ± 18.7	131.3 ± 23.3	123.4 ± 13.1
Diastolic blood pressure (mm Hg)	81.0 ± 9.0	75.0 ± 10.4	77.0 ± 19.9	78.6 ± 11.0
Current smokers (%)	6.3	18.2	2.1	25.0
Preexisting diabetes (%)	0.0	4.5	10.2	0.0
Preexisting heart disease (%)	0.0	5.9	0.8	2.0
Preexisting cancer (%)	0.0	7.1	13.7	2.0

Demographic and clinical characteristics of the cohorts used in the present study are presented. Values are numbers of patients, percentages (%), mean ± standard deviation, or median ± median absolute deviation, as appropriate.

We investigated whether the predictive ability of glucuronic acid for mortality would be attenuated by including other known mortality biomarkers in the regression model. The Framingham Offspring cohort allowed us to correct for seven of the most commonly studied biomarkers of mortality, including fasting glucose, HDL cholesterol, LDL cholesterol, triglycerides, creatinine, HbA1c, and albumin. In a multivariate Cox regression model adding these to our baseline clinical covariates, glucuronic acid levels remained a significant predictor of mortality (HR=1.12, p=0.044).

### Circulating glucuronic acid levels predict mortality across species

To further validate the observed relationship between glucuronic acid levels and mortality, we performed metabolomic profiling on the sera of 196 27-month-old female mice from a genetically heterogenous background followed from birth through 33 months of age. In this murine cohort, glucuronic acid levels were also a significant predictor of all-cause mortality (HR=1.29, p=0.018), and a weakly significant difference in survival was observed between the top and bottom quartiles of glucuronic acid levels in this cohort (p=0.1, Figure 1C).

### Circulating glucuronic acid levels increase with age

**Figure 2:**
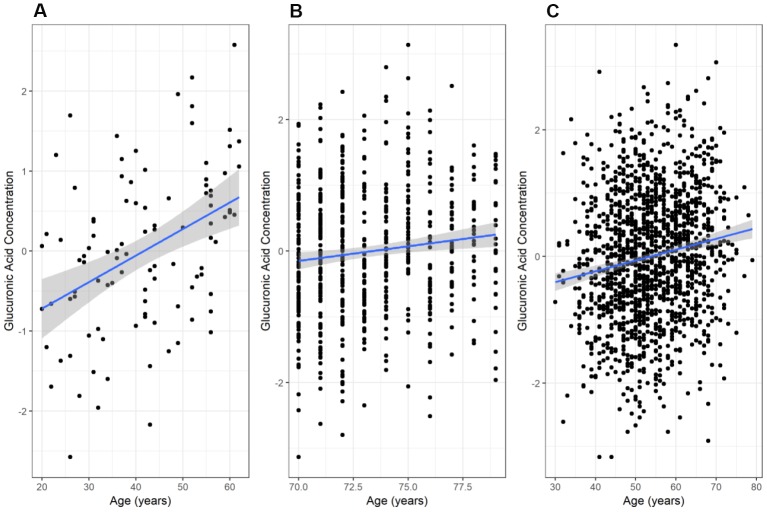
**Variation of glucuronic acid levels with age.** The relationship between chronological age and glucuronic acid levels in (**A**) the Estonian Biobank secondary cohort, (**B**) the Estonian Biobank primary (discovery) cohort, and (**C**) the Framingham Offspring cohort. In each case, the best-fit regression line through the data is shown, with the 95% confidence interval for this line shaded in grey. Glucuronic acid concentrations are represented as standard deviations of normalized concentrations within each dataset.

We found a significant positive correlation between glucuronic acid levels and age in the secondary Estonian Biobank cohort (N=100; r=0.41, p=2.7×10^-5^, Figure 2A), which was selected to span a wide range of ages. This relationship also validated in the main Estonian Biobank cohort (Pearson r=0.12, p=3.7×10^-3^, Figure 2B) and the Framingham Offspring cohort (r=0.16, p=1.3×10^-9^, Figure 2C). Multivariate models adjusted for standard clinical covariates also identified age as a significant correlate of glucuronic acid levels in all three cohorts (p=4.3×10^-3^, 2.0×10^-8^, and 2.2×10^-5^, respectively).

### Circulating glucuronic acid levels predict healthspan-related outcomes

We sought to further elucidate the predictive ability of glucuronic acid for age-related decline in health. In the Framingham Offspring metabolomics cohort, elevated levels of glucuronic acid were associated with poorer healthspan-related outcomes measured more than a decade in the future, including reduced overall self-rated health (p=0.11), reduced grip strength (p=0.027), reduced self-rated ability to perform heavy housework (p=0.023), higher measured times on tests of normal walking speed (p=0.018) and quick walking speed (p=9.4×10^-4^), as well as reduced pulmonary forced expiratory volume in one second (FEV1; p=0.046), a measure of lung function (Table 2). In general, an increase of glucuronic acid levels by one standard deviation corresponded to approximately the same functional decline and mortality risk expected from an additional year of age.

**Table 2:** Regression results relating glucuronic acid concentration to future healthspan-related outcomes.

**Phenotype**	**Regression model**	**Regression coefficient**	**Units**	**P-value**
Self-rated health	Ordinal	-0.0837	Points / 1 SD	0.0106 *
Grip strength	Linear	-0.397	Kg / 1 SD	0.0271 *
Walk time	Linear	0.0539	Min / 1 SD	0.0184 *
Quick walk time	Linear	0.0576	Min / 1 SD	0.000943 ***
FEV1	Linear	0.0291	L / 1 SD	0.0463 *
Housework capability	Logistic	-0.175	Good vs. Poor	0.0233 *

Linear, logistic, or ordinal regression models were fit to determine the predictive association of glucuronic acid with each phenotype. Analyses were corrected for standard clinical covariates. Self-rated health was measured on 5-point scale; grip strength was measured in kilograms; walk time and quick walk time were measured in minutes; FEV1 (forced expiratory volume in 1 second) was measured in liters; housework capability was measured as a binary outcome. Units for the regression coefficient are given to the right of the regression coefficient values, and correspond to the above test-specific units per 1 standard-deviation change in concentration of glucuronic acid.

## DISCUSSION

We have demonstrated that glucuronic acid levels are robust predictors of all-cause mortality and correlate with future healthspan-related outcomes. The effect size of the relationship with mortality (hazard ratios between 1.1 and 1.4 in our cohorts) is comparable to that of existing, clinically-important biomarkers of mortality such as cholesterol (HR=1.12 per mmol/L increase) and systolic blood pressure (HR=1.13 per 10 mm Hg increase) [32, 33]. Importantly, the predictive utility of glucuronic acid persists after adjustment for standard clinical covariates and other accepted predictors of mortality, including factors such as demographics, BMI, smoking status, blood lipids, HbA1c, creatinine, and albumin, indicating that the predictive ability of glucuronic acid for mortality is independent of these existing markers and their related biological mechanisms [34]. Moreover, we have demonstrated novel associations between glucuronic acid levels and future healthspan-related outcomes, including physical abilities, functional capabilities, and self-rated health, suggesting that the risk of mortality associated with elevated glucuronic acid levels is accompanied by a general decline in healthspan. Finally, we found glucuronic acid to be strongly positively correlated with age in three human cohorts with mean age ranging from 40 to 70 years (Table 1), an association that remained statistically significant following adjustment for clinical covariates. Notably, the simultaneous association of glucuronic acid levels with age, lifespan (as determined by all-cause mortality), and healthspan-related outcomes strongly argues that glucuronic acid is a biomarker of biological aging. Depending on the specific biology that underlies this relationship, glucuronic acid may also relate to the pathogenesis of these outcomes, and hence also of biological aging.

There are multiple mechanisms that might link levels of circulating glucuronic acid to age, mortality, and healthspan. One of the most compelling and best-understood possibilities relates to the cleavage of glucuronic acid from glucuronidated xenobiotics and steroid hormones by intestinal bacteria, a process that releases glucuronic acid as well as the toxin or steroid, making both available for reabsorption into the bloodstream in a process called enterohepatic recirculation [10]. Since activity of the responsible enzyme, β-glucuronidase, varies greatly among microbial species [12, 35], the composition of a person’s intestinal microbiome has a direct influence on this process. A microbiome that rapidly cleaves glucuronide conjugates may thus produce elevated glucuronic acid and also interfere with xenobiotic elimination and steroid metabolism. The substrates of glucuronide conjugation include xenobiotics such as environmental toxins and drug metabolites with pro-inflammatory and immunosuppressive effects [36–38] as well as endogenous steroids with tumorigenic effects at high concentrations [39, 40], and impairment of glucuronidation by intestinal β-glucuronidase can cause organ toxicity, inflammatory disorders, and carcinogenesis [16, 41–44]. Consequently, changes in microbiome composition could easily underlie the observed correlation between higher glucuronic acid levels, age, healthspan-related outcomes, and mortality [45].

This hypothesis is supported by other evidence from the literature. Among older people, bacterial β-glucuronidase activity levels are increased relative to young people (Mroczyńska and Libudzisz, 2010), and microbiome differences can distinguish healthy, independent older people from those who tend to be frail, sick, and require long-term residential care [46, 47]. Microbiome composition has also been linked to the onset of numerous age-related diseases, including atherosclerosis [48], type 2 diabetes [49], Alzheimer’s disease [50], chronic kidney disease [51], and nonalcoholic steatohepatitis [52], all of which contribute to reduced healthspan and increased mortality. Furthermore, bacterial β-glucuronidase activity changes in the appropriate direction with dietary modifications. For example, red meat consumption alters the gut microbiome [53, 54], elevates fecal β-glucuronidase activity [55], and correlates with increased future diabetes, cardiovascular disease, and mortality [56]. In contrast, dietary fiber consumption increases microbial diversity [57], reduces fecal β-glucuronidase activity [58–60], and correlates with lower all-cause and cause-specific mortality [61–63].

While this hypothesis is compelling, many other possibilities exist. For example, bacterial β-glucuronidase activity may be affected by gastrointestinal pH [64, 65] and hepatic glucuronidation may be impaired by chronic renal failure [66], both of which are independent of microbiome composition. Another potential mechanism linking glucuronic acid levels to disease states involves endogenous human β-glucuronidase, which localizes primarily to the lysosome and degrades glycosaminoglycans during normal and pathologic remodeling of the extracellular matrix (ECM) via hydrolytic liberation of glucuronic acid [21]. ECM remodeling is increased in aging and age-related diseases [67–69], with ECM degradation fragments in serum even being employed as disease biomarkers in some cases [70–72]. Moreover, lysosomal membrane permeabilization, an observation to inflammation and cell death [73–79] can cause the release of endogenous β-glucuronidase into the bloodstream [80], where it cleaves glucuronidated conjugates and may contribute to circulating glucuronic acid levels [81, 82]. In these scenarios, elevated glucuronic acid levels could be a result of ECM remodeling, inflammation, or cell death caused by concurrent disease. This seems less likely in our discovery cohort, where participants were free of major diseases at sample collection, but could still be consistent with subclinical disease. Finally, in addition to the above mechanisms related to glucuronidation, glucuronic acid may directly elicit an inflammatory response through an interaction with toll-like receptor 4 (TLR4) [83], which has been implicated in the pathophysiology of age-related diseases [84–86]. Of course, substantial further research is warranted to distinguish the relative contributions of these various hypotheses to the link between circulating glucuronic acid levels and aging, healthspan, and mortality.

The present study has a number of limitations. First, it is observational and retrospective in nature; however, it derives strength from the persistence of association across three high-quality, prospective human cohort studies in different countries, as well as in a lifespan study of genetically heterogenous mice. Second, despite the geographic diversity of our cohorts (Estonia, USA, and the United Kingdom), study participants were still predominantly of European descent, and the generalizability of these results to other demographic groups is uncertain, although the positive cross-species replication is encouraging. Third, the human cohorts were under-powered to evaluate relationships of glucuronic acid with the incidence of most individual diseases.

In summary, circulating glucuronic acid levels predict mortality in humans and mice, and in humans also associate with chronological age and predict healthspan-related outcomes. These simultaneous associations with age and with factors defining both lifespan and healthspan provide strong evidence that glucuronic acid is a biomarker of longevity and healthspan, as well as underlying biological age.

## MATERIALS AND METHODS

### Sample cohorts

### Estonian Biobank cohorts

Our discovery cohort was drawn from the Estonian Biobank at the University of Tartu [87]. Participants were selected to be elderly (between the ages of 70 and 79) and healthy at the time of sample collection, i.e. to have no preexisting history of hypertensive heart disease, diabetes, coronary artery disease, cancer, chronic obstructive pulmonary disease, stroke, or Alzheimer’s disease. The resulting 579 participants have 8 to 14 years of clinical follow-up for mortality (187 deaths observed). A secondary cohort of 100 members was independently selected from the Estonian Biobank to span a wide range of ages (20-60 years) at the time of sample collection.

### Framingham cohort

The Framingham Offspring cohort includes children of the original Framingham Heart Study participants, recruited in 1971 [88]. Of the entire cohort, 1,479 participants in the fifth examination cycle (between 1991 and 1995) consented to both metabolomic profiling and broad research use of their samples. These form the Framingham Offspring metabolomic cohort used in this study [89], with an age range of 30 to 79 years at blood draw and 30-40 years of clinical follow-up (232 deaths observed).

### TwinsUK cohort

The TwinsUK cohort used in this study comprises the 1,761 individuals for whom glucuronic acid level measurements and matching covariate measurements were available [90], with a mean age of 64.9 ± 8.4 years at blood draw and approximately 5 years of clinical follow-up (47 deaths observed).

### BIOAGE mouse study

300 female mice were bred by Vium (San Mateo, CA, USA) as a four-way cross among DBA/2J, C3H/HeJ, BALB/cJ, and C57BL/6J mice purchased from Jackson Laboratories (Sacramento, CA, USA). The mice were housed with Vium from birth onwards and allowed to age naturally without interventions other than regular weighing, blood collection, and humane euthanasia. For metabolite profiling, approximately 150 uL of whole blood was collected via submental (primary) or submandibular (secondary) technique from each of the 196 mice still surviving at 27 months of age. Collected blood was allowed to clot without additive and centrifuged, and serum was subsequently extracted. Mice continued to be monitored through 33 months of age, with 106 recorded deaths occurring after the blood draw.

### Metabolite profiling

For the Estonian Biobank cohorts, non-fasting plasma samples were collected from each participant at enrollment and stored in liquid nitrogen, then shipped to the Broad Institute (Cambridge, MA, USA) for metabolomic profiling via liquid chromatography-mass spectrometry (LC-MS) as previously described [91]. Briefly, metabolites were extracted from plasma samples with four methods: (1) amines and polar metabolites were extracted with a mixture of acetonitrile and methanol and separated with a HILIC column under acidic mobile phase conditions; (2) central metabolites and negatively ionizing polar metabolites were extracted using 80% methanol and separated with a HILIC column under basic conditions; (3) free fatty acids, bile acids, and metabolites of intermediate polarity were extracted using 100% methanol and separated with reverse phase chromatography on a T3 ULPC column; lastly, (4) lipids were extracted using 100% isopropanol and separated with reverse phase chromatography on a C4 column. For the Framingham Offspring metabolomics cohort, fasting plasma samples were collected from participants during the fifth examination cycle and stored at -80°C until metabolite profiling as previously described (Wang et al., 2011; dbGap ID phs000007.v30.p11), comprising of the targeted identification and quantification of 217 metabolites with liquid chromatography-tandem mass spectrometry. For the TwinsUK metabolomics cohort, fasting serum samples were collected from participants and stored at -80°C until analysis using ultra-high performance liquid chromatography-tandem mass spectrometry by Metabolon, Inc. (Research Triangle Park, NC, USA) as previously described [92]. For the murine cohort, whole blood samples were drawn at day 800 of the study; serum was prepared as described above and sent to the West Coast Metabolomics Center (Davis, CA, USA) for untargeted metabolomics profiling via GC-TOF MS/MS.

### Statistical analyses

Raw metabolite intensity values were corrected for instrumental drift and underwent rank inverse normal transformation [93]. Missing covariate data were imputed as the dataset mean, and final study results underwent sensitivity analysis to demonstrate that the imputation did not cause substantive changes. Hazard ratios and p-values for all-cause mortality were determined by fitting a Cox proportional hazards model with each metabolite (e.g. glucuronic acid) as the main predictor and including as clinical covariates age, sex, smoking status, body mass index, systolic blood pressure, and diastolic blood pressure [94]. Kaplan-Meier survival curves for the upper and lower quartiles of glucuronic acid level were plotted and the log-rank test was used to test for differences between the curves. In the TwinsUK cohort, analysis also took into account family relatedness. Validation analyses for mortality in the Framingham Offspring and TwinsUK cohorts were performed using one-sided statistical tests; all other comparisons used two-sided tests.

Correlations between glucuronic acid levels and age were calculated using Pearson’s r. Multivariate linear regression was used to calculate the association, corrected for clinical covariates, of glucuronic acid levels with age, grip strength, walking speed, and forced expiratory volume in 1 second (FEV1), respectively. Multivariate logistic regression was used to calculate the association of glucuronic acid levels with housework capability, corrected for clinical covariates. Multivariate ordinal regression via a cumulative link model was used to calculate the association of glucuronic acid levels with subjective overall health, corrected for clinical covariates [95]. In all cases, clinical covariates were selected as in the mortality analyses. The healthspan-related phenotypes above were measured in the eighth examination cycle for the Framingham Offspring cohort. All statistical analyses and generation of graphics were performed with R version 3.3.3 [96].
